# Complete genome sequences of toxigenic *Clostridioides difficile* isolated from Australian feral horses

**DOI:** 10.1128/mra.01086-24

**Published:** 2024-11-22

**Authors:** Natasza Hain-Saunders, Daniel R. Knight, Mieghan Bruce, Thomas V. Riley

**Affiliations:** ^1^Centre for Biosecurity and One Health Harry Butler Institute, Murdoch University, Murdoch, Western Australia, Australia; 2 School of Biomedical Sciences, The University of Western Australia, Queen Elizabeth II Medical Centre, Nedlands, Western Australia, Australia; 3 Department of Microbiology, PathWest Laboratory Medicine, Queen Elizabeth II Medical Centre, Nedlands, Western Australia, Australia; 4 School of Medical and Health Sciences, Edith Cowan University, Joondalup, Western Australia, Australia; Rochester Institute of Technology, Rochester, New York, USA

**Keywords:** *Clostridium difficile*, genomics, horse, CDI, *Clostridioides difficile*

## Abstract

Recent increases in community-associated *Clostridioides difficile* infections have highlighted the importance of monitoring toxigenic *C. difficile* from animal and environmental sources. We provide the complete circularized genomes of two toxigenic *C. difficile* strains isolated from feral horse faeces. Genome N64 (sequence type 964) consists of a single chromosome of 4,078,791 bp, while genome H251 (sequence type 963) comprises one chromosome (4,304,722 bp) and three plasmids (150,942 bp, 11,534 bp, and 9,074 bp).

## ANNOUNCEMENT

*Clostridioides difficile* is an anaerobic bacterium and a major cause of diarrhea and life-threatening enteric disease in both animal and human populations. With community-associated disease on the rise (1, 2), surveillance of emerging toxigenic strains from nontraditional sources is becoming increasingly important. Here, we provide complete and circularized genomes for two toxigenic strains of *C. difficile* (NT64 and H251) isolated from Australian feral horses.

Feral horse fecal samples were collected from the Northern Territory (NT64) in 2020 and New South Wales (H251) in 2017. Collection sites were identified in collaboration with Brumby associations, agricultural landowners and traditional landowners based on known population territories and reports of recent sightings. H251 was collected as part of a population ecology, health, and welfare survey (3).

Fecal samples were cultured for *C. difficile* using previously described enrichment methods (4). DNA was extracted using the QuickGene DNA DT-S tissue kit as per the manufacturer’s instructions (Kurabo Industries, Osaka, Japan). Short-read genome sequencing was performed on the Illumina NovaSeq 6000 platform, with Nextera Flex 150-bp paired-end chemistry to a depth of coverage of 145X (Illumina, San Diego, CA, USA). Long-read sequencing utilized the NanoPore MinION Mk1C platform (Oxford Nanopore Technologies, Oxford, UK), with sequencing libraries prepared using the Ligation Sequencing Kit (SQK-LSK109) and run on a FLO-MIN106 flow cell for 12 hours.

Both short and long sequence reads were trimmed with TrimGalore v0.6.7 (https://github.com/FelixKrueger/TrimGalore). Long reads were additionally filtered using Filtlong v0.2.0 (https://github.com/rrwick/Filtlong) to remove low-quality reads of less than 1,000 base pairs. Hybrid assembly of short-read and long-read data was performed using Unicycler v0.5.0 (in the conservative mode [5]), followed by multiple rounds of polishing (pilon v1.2.4, racon v1.4.3) to improve contiguity. Complete circularised genomes were confirmed using Bandage v0.9.0 (6) and rotated to *dnaA* using Unicycler. Genome annotation was performed using the NCBI Genome Annotation Pipeline v5.2 (7), and sequence data were submitted to GenBank under BioProject PRJNA858229 (accession CP101707) and PRJNA865080 (accessions CP102400, CP102401, CP102402 and CP102403). The multilocus sequence type (ST) of strains was determined using PubMLST (BIGSdb v1.32.0) (8). Prophages were characterized using PHASTER (9). Abricate v1.0 (https://github.com/tseemann/abricate) was used to screen for AMR genes (using the NCBI database) and *C. difficile* toxin genes *tcdA*, *tcdAB,* and *cdtA/B* (using a custom database built from published *C. difficile* reference genomes). All analysis software were set to default parameters, unless otherwise stated.

Analysis of strains N64 and H251 reported a whole-genome length of 4,078,791 and 4,304,722 bp and GC content of 28.22 and 28.48, respectively. Three additional extrachromosomal elements were identified for H251 (150,942 bp, 11,534 bp, and 9,074 bp). Information on the assembled chromosomes and extrachromosomal elements is shown in Table 1.

**TABLE 1:** Key features of *C. difficile* genomes isolated from Australian feral horse feces

Features	Isolate	
	NT64	H251
Origin	Feral horse Northern Territory, 2020	Feral horse, New South Wales, 2017
PCR ribotype	Novel	Novel
Multi-locus sequence type	964 (Clade 5)	963 (Clade 4)
Toxin profile^a^	A – B – CDT+	A + B + CDT+
Chromosome		
GenBank accession	CP101707	CP102400
Size (bp)	4,078,791	4,304,722
N contigs	1	1
%GC	28.22	28.48
N CDS	3,685	3,992
N tRNA/rRNA	90/35	90/35
N CRISPRs^b^	4	7
Prophages	vB CpeS-CP51	ΦSM101; CDKM15; ΦCT19406A
AMR loci	*bla*CDD-2; vanZ1	*bla*CDD-2
Extrachromosomal elements (GenBank accession)		
Novel plasmid 1	Nil	150,942 bp (CP102401)
Novel plasmid 2	Nil	11,534 bp (CP102402)
Novel plasmid 3	Nil	9,074 bp (CP102403)
Illumina reads		
Total reads (trimmed)	2,150,427	2,056,855
SRA accession number	SRR26799532	SRR26799533
ONT reads		
Total reads (filtered)	518,419	240,315
SRA accession number	SRR26800611	SRR26800676

^a^ Toxin profile: presence/absence of full-length *tcdA* (A), *tcdB* (pathogenicity locus) (B), and binary toxin *cdtA/B* (binary toxin locus) (CDT).

^b^ CRISPRs, clusters of regularly interspaced short palindromic repeats.

## Data Availability

Complete genome assemblies for NT64 and H251 are available in GenBank under BioProject numbers PRJNA858229 (accession numbers CP101707) and PRJNA865080 (accession numbers CP102400, CP102401, CP102402, and CP102403). Sequence read data are available in the NCBI Sequence Read Archive (SRA) with Illumina reads under SRA accession numbers SRR26799532 (NT64) and SRR26799533 (H251); and NanoPore reads under SRA accession numbers SRR26800611 (NT64) and SRR26800676 (H251).
